# Iterative Image Reconstruction for Limited-Angle CT Using Optimized Initial Image

**DOI:** 10.1155/2016/5836410

**Published:** 2016-01-26

**Authors:** Jingyu Guo, Hongliang Qi, Yuan Xu, Zijia Chen, Shulong Li, Linghong Zhou

**Affiliations:** School of Biomedical Engineering, Southern Medical University, Guangzhou 510515, China

## Abstract

Limited-angle computed tomography (CT) has great impact in some clinical applications. Existing iterative reconstruction algorithms could not reconstruct high-quality images, leading to severe artifacts nearby edges. Optimal selection of initial image would influence the iterative reconstruction performance but has not been studied deeply yet. In this work, we proposed to generate optimized initial image followed by total variation (TV) based iterative reconstruction considering the feature of image symmetry. The simulated data and real data reconstruction results indicate that the proposed method effectively removes the artifacts nearby edges.

## 1. Introduction

Computed tomography (CT) has been applied extensively in clinical diagnosis. Under some CT applications, limited-angle scan mode has deserved wide interest [1] since it has to be used in several practical cases such as large object size [2], short exposure time [3], and restricted scanning [4]. Limited-angle scan means that projection data are obtained in less than 180° angular range when X-ray tube and detector rotate around the scanned object.

CT image reconstruction algorithms can be classified into analytical reconstructions and iterative reconstructions. Filtered back projection (FBP) reconstruction [5], as a representative of analytical methods, needs a high sampling rate of projection data to reconstruct satisfactory images. However, when FBP is chosen, incomplete projection data due to limited-angle scan often generate bad results with severe artifacts, which affect accurate diagnosis. In the past decades, studies have found that iterative reconstructions have potential to suppress image artifacts under a limited-angle scan mode. Advanced iterative algorithms have been developed, such as singular value decomposition [6], wavelet decomposition [7], and projections onto convex sets (POCS) [8]. However, these methods cannot well solve edge distortions, although smooth artifacts nearby edges are effectively reduced. Taking advantage of prior information is another interesting path to study limited-angle reconstruction. Priors relevant to boundary, shape, density range, and so forth have been successfully added to CT image reconstruction algorithms. Candes et al. indicated that medical images by taking the gradient transform are sparse and proposed compressed sensing (CS) reconstruction theory [9, 10]. Some exemplary algorithms based on CS have been reported to provide good reconstruction results in the case of sparse-view or few-view reconstruction [11–17]. The most classical algorithm is POCS with TV minimization (POCS-TV) proposed by Sidky et al. [18, 19]. For limited-angle CT, POCS-TV reconstructs better images than conventional algebraic reconstruction technique (ART) [20] and maximum likelihood expectation maximization (MLEM) [21]. However, POCS-TV and other TV based methods still cannot produce satisfactory results, and large amount of iterations is needed to make the reconstruction converge.

Utilizing the prior information plays a vital role in reconstructing high-quality images for limited-angle CT. However, the existing iterative methods do include priors inside the main iteration body, while initialization was overlooked [17, 18]. After such common initializations, a large number of iterations are needed to produce relatively acceptable image and apparent artifacts nearby edges still can be noticed. To our knowledge, study on choosing optimal initial image for iterative reconstruction has not been published before. Therefore, how to get optimal initial image for limited-angle CT reconstruction is our priority in this work.

In some specific applications, outer contours of some reconstructed objects are roughly axis-symmetrical, for example, head imaging and industry component imaging. The symmetry axis is often located in the central row (or column) of an image, if the scanned object is placed properly. Additionally, the structures surrounding object's outer contour are also roughly axis-symmetrical. Based on the observation, we propose an iterative image reconstruction algorithm with optimized initial image for limited-angle CT. Using the property of object's outer contour axis-symmetry, the local artifacts in image reconstructed by FBP can be removed. Then, the artifacts-removed image is regarded as initial image for iterative reconstruction (POCS-TV is applied in this paper). Results from different initial images (zero image, FBP image, and our optimized image) for POCS-TV are compared. The efficacy of the proposed method was assessed by simulations on the Shepp-Logan phantom and realistic head phantoms. We addressed limited-angle artifacts in POCS-TV, already evidenced in the cited paper [18], proposing a novel initialization procedure. Benefits are foreseen to all iterative algorithms, but their assessment is beyond our scope.

The rest of the paper is organized as follows. In the next section, the contour symmetry and mirroring filling method is described, and the POCS-TV method is summarized. In the third section, experimental results are presented and discussed.  Section 4 concludes the paper.

## 2. Method

### 2.1. Imaging Geometry

**Figure 1:**
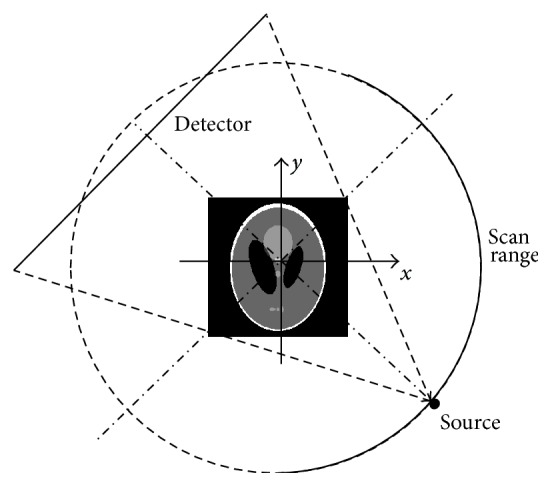
Fan-beam limited-angle CT geometry configuration.

The imaging geometry for fan-beam limited-angle CT is illustrated as in Figure 1. We choose a circular scanning locus. In the experiments of this paper, the initial position of X-ray source is located in −*y*-axis and the X-ray source does a counterclockwise limited-angle rotation (such as 150° and 120°) to collect projection data evenly. Finally, we assume that the object to be scanned is put properly so that the outer symmetrical axis parallels *y*-axis.

In such an imaging geometry, the image reconstructed by traditional methods (such as FBP) contains severe artifacts and most of them are located in the second and forth quadrants. The results can be seen in the experiments from Section 3.

### 2.2. Contour Symmetry and Mirroring Filling

**Figure 2:**
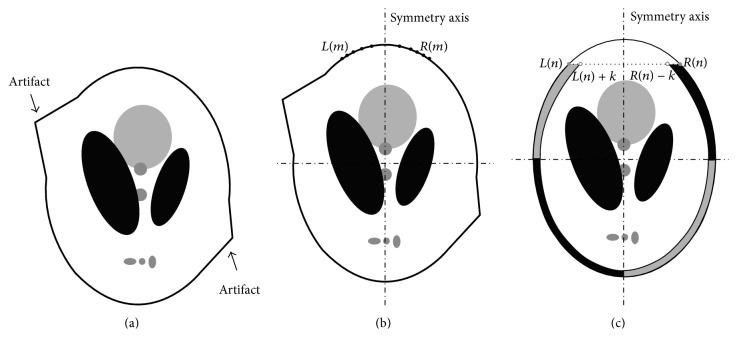
Illustration of calculating the symmetry axis and mirroring filling operation: (a) FBP image model with artifacts, (b) calculating the symmetry axis, and (c) mirror filling operation to remove artifacts.

From many medical CT images, we have observed that some of objects' outer contours are roughly axis-symmetrical and the symmetry axes are parallel with columns (or rows) of images, if the objects are suitably positioned. Additionally, the information surrounding object's outer contour is also roughly axis-symmetrical. Although the reconstructed image under the scanning modality described in Figure 1 contains severe artifacts in the second and forth quadrants (Figure 2(a)), it is possible to cancel most of the artifacts by using the information from the first and third quadrants in the image.

The method of calculating the symmetry axis of outer contour in an image can be described as follows. First, the image of object is reconstructed by FBP in which the projection data with zero values is used to set the pixels out of the object to be zero. The above process facilitates the positioning of object's contour. Then, we search for the pixels row by row until the contour is detected. The left contour point and right contour point are noted as *L*(*m*) and *R*(*m*) and *m* is the row index. More *J* rows (*J* = 10 in this paper) are needed to search for the contour points and the set of *L*(*m* + *j*) and *R*(*m* + *j*)  (*j* = 0,1, 2,…, *J*) areobtained to calculate symmetry axis more accurately.  Figure 2(b) illustrates the procedures of locating symmetry axis. The expression of calculating symmetry axis is defined as

(1) $$\begin{array}{c} S=\frac{1}{J+1}\overset{J}{\underset{j=0}{\sum }}\frac{\left(R\left(m+j\right)+L\left(m+j\right)\right)}{2}. \end{array}$$

The method of canceling artifacts is described as follows. Suppose an image with *N* rows and *M* columns. For the upper half of the image, the first quadrant is used to fill up the artifacts from the second quadrant. For *n*th row, *n* = *m* + *J*,…, *N*/2, the right contour point *R*(*n*) can be found. According to the symmetry axis *S*, the left contour point *L*(*n*) can be calculated. The pixels which are at *L*(*n*) point's left side are set to be zero. Meanwhile, the pixels between *R*(*n*) and *R*(*n*) − *K* (the black area in Figure 2(c)) are reflected to pixels between *L*(*n*) and *L*(*n*) + *K* (the gray area in Figure 2(c)) in the mirror. For the lower half of the image, the artifacts can be removed in the same manner. Illustration of mirroring filling operation is shown in Figure 2(c).

### 2.3. The Proposed Reconstruction Method for Limited-Angle CT

The symmetry is employed to produce an image with a smart reduction of artifacts. The image serves as initialization for iterative reconstruction. Classical POCS-TV method is applied to perform iterative reconstruction in this paper. Implementation of the POCS-TV algorithm [18] using the proposed initialization is summarized as follows.

**Step 1 (perform the initialization procedure).**

Define initial image as *f* = (*f*_1_, *f*_2_,…,*f*_*N*_)^*T*^. *N* is total number of image pixels. Set *f* ^ART^[*k*, 0] = *f* and *k* = 1, 2,…, *K* denotes iteration index.

**Step 2 .**

ART reconstruction is as follows:

(2) fARTk,m=fARTk,m−1+Aiyi−Ai·fARTk,m−1Ai·AiT,

where *y* = (*y*_1_, *y*_2_,…,*y*_*M*_)^*T*^ denotes projection data. *m* = 1,2,…, *M* and *M* is total number of X-ray beams. *A* = {*a*_*ij*_} is the system matrix with size of *M* × *N* which accounts for the system geometry. *a*_*ij*_ represents the length of the intersection of the X-ray *i* with the pixel *j*.

**Step 3 .**

Positivity constraint is as follows:

(3) $$\begin{array}{c} f_{j}^{\left(\text{TV-POS}\right)}\left[\;k\;\right]=\left\{\begin{array}{cc} f_{j}^{\left(\text{ART}\right)}\left[k\right] & f_{j}^{\left(\text{ART}\right)}\left[k\right]\geq 0\\ 0 & f_{j}^{\left(\text{ART}\right)}\left[k\right]<0. \end{array}\right. \end{array}$$

**Step 4 .**

TV minimization using gradient steep algorithm is as follows:

(4) fTV-GRADk=TV_minimizationfTV-POSk,fARTk+1=fTV-GRADk.

**Step 5 .**

Return to Step 2 until the stopping criterion is satisfied.

## 3. Numerical and Experimental Studies

To assess the effectiveness of our proposed method, Shepp-Logan phantom and real head phantom are used for limited-angle CT reconstruction. Both qualitative and quantitative studies on the results are conducted.

### 3.1. Numerical Simulation

**Figure 3:**
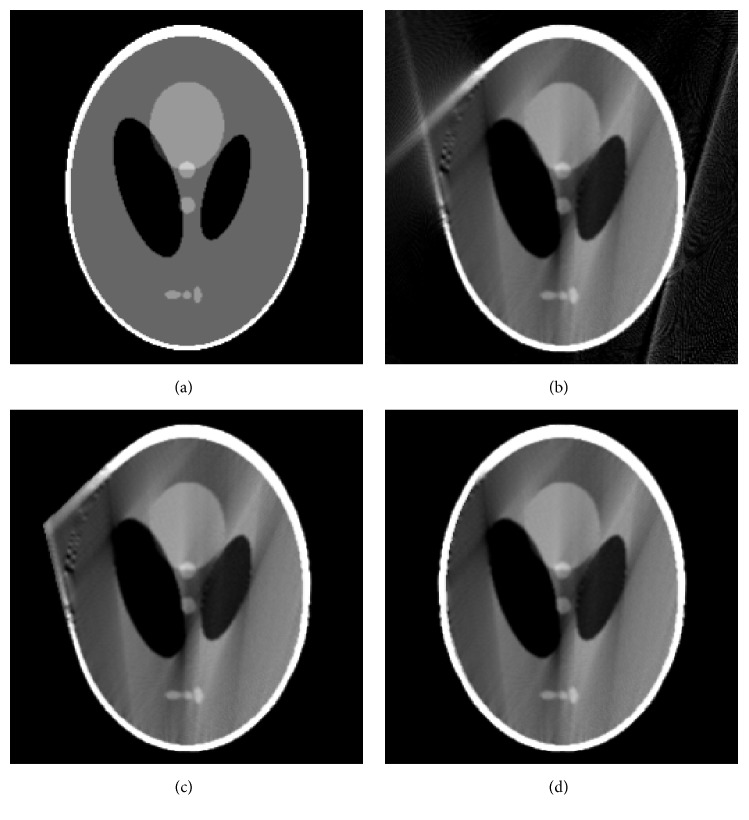
Shepp-Logan phantom and initial images: (a) phantom, (b) reconstructed image using FBP method, (c) reconstructed image using both FBP method and air correction, and (d) reconstructed image using the proposed algorithm.

In this section, we conduct numerical studies to evaluate the performance of POCS-TV with different initial images (zero image, FBP image, and our optimized initialization). The size of Shepp-Logan phantom image is 256 × 256. The distance between X-ray source and rotating axis is 40 cm and the distance between detector and rotating axis is 40 cm. The detector is a line array consisting of 512 elements. The initial position of X-ray source is located in −*y*-axis and the X-ray source does a counterclockwise rotation, as illustrated in Figure 1. A total of 150 projections are equally captured over a 150-degree range. In our experiments, 50 iterations for POCS-TV are sufficient for stable results. The tests in this paper are implemented by MATLAB programming language on a PC with Intel(R) Core(TM) 2 Quad CPU 2.50 GHz and 3.25 GB RAM. True Shepp-Logan phantom, image reconstructed by conventional FBP, image reconstructed by FBP with air correction technique, and image reconstructed by our proposed initialization procedure are shown in Figures 3(a)–3(d), respectively. It is clear to see that Figure 3(b) has severe artifacts.  Figure 3(c) contains fewer artifacts due to air correction technique, but geometric distortions exist conspicuously. Conversely, our proposed method leads to least artifacts and geometric distortions shown in Figure 3(d), which demonstrates that Figure 3(d) would be the optimal initial estimation for iterative POCS-TV reconstruction.

**Figure 4:**
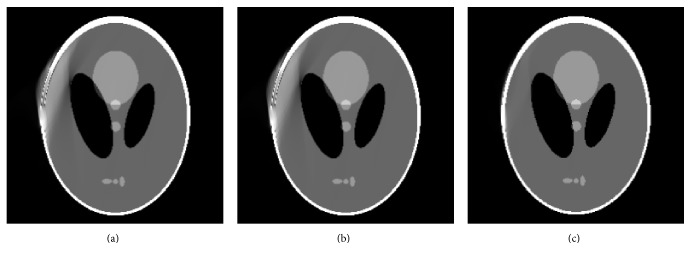
Reconstruction results by different initial images: (a) result from POCS-TV with initial zero image, (b) result from POCS-TV with initial FBP image, and (c) result from POCS-TV with proposed initial image.

The images reconstructed by POCS-TV choosing zero image, FBP image (Figure 3(b)), and our optimized image (Figure 3(d)) as initializations are shown in Figure 4. Reconstructions (Figures 4(a) and 4(b)) from POCS-TV with initial zero image and FBP image produces unsatisfactory results with the artifacts not removed effectively. Visually, Figures 4(a) and 4(b) have the same image quality. As shown in Figure 4(c), POCS-TV with our optimized initial estimation can reconstruct high-quality image in which artifacts are effectively suppressed and edge structure information is better preserved.

In addition to visual inspection of the results, the mean square error (MSE) and signal to noise ratio (SNR) measures are used. The definitions of SNR and MSE are listed as follows:

(5) MSE=1N∑iNfi∗−fi2,SNR=10 log10⁡∑iNfi−f_aver2∑iNfi∗−fi2,

where *f*_*i*_ denotes the image reconstructed, *f*_*i*_^*∗*^ denotes the original phantom image, *N* is the total number of image pixels, and *f*__aver_ denotes the average value of the reconstructed image.

**Figure 5:**
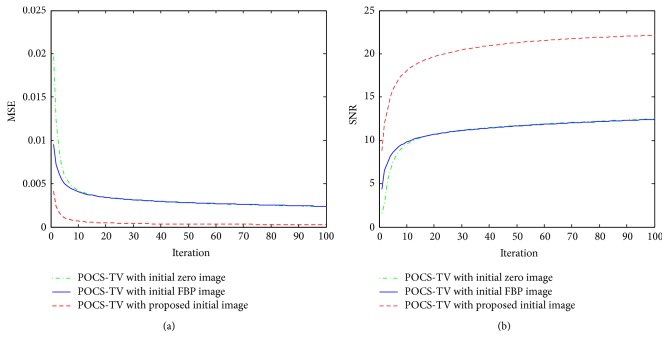
MSE and SNR curves from different initial image: (a) MSE curves and (b) SNR curves.

Figure 5 presents the MSE and SNR curves with the iteration number increases. The quantitative results from POCS-TV with the proposed initialization exhibit lower MSE and higher SNR than POCS-TV with zero image and FBP image. It demonstrates that our method can produce stable and high-quality image using less iteration numbers.

Practically, the outer contour of an object is not accurately axis-symmetrical. To verify the effectiveness of our proposed method, we modify Shepp-Logan phantom to be asymmetrical. Two experiments in this case are studied.

**Figure 6:**
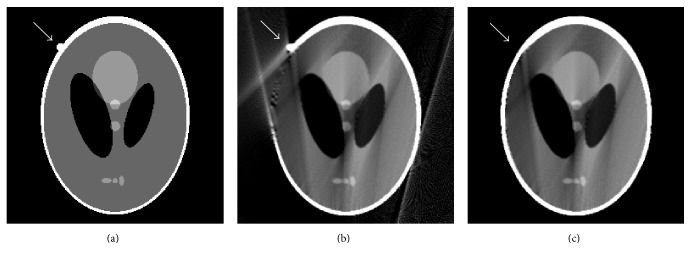
Modified Shepp-Logan phantom and initially reconstructed images: (a) modified phantom, (b) reconstructed image by FBP, and (c) reconstructed image using the proposed algorithm.

**Figure 7:**
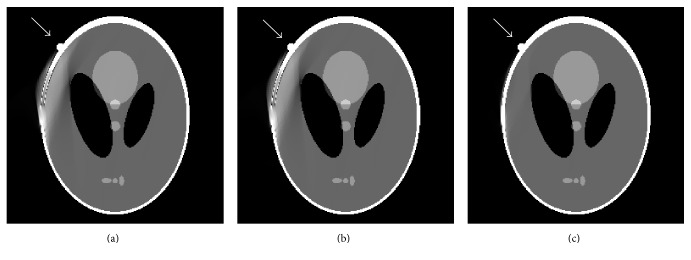
Reconstruction results by different initial images: (a) result from POCS-TV with initial zero image, (b) result from POCS-TV with initial FBP image, and (c) result from POCS-TV with proposed initial image.

The modified Shepp-Logan is shown in Figure 6(a). One small circle tissue is added. Initial images reconstructed by conventional FBP and our proposed method are shown in Figures 6(b) and 6(c), respectively. It illustrates that there exist severe artifacts and geometric distortions in Figure 6(b). Due to mirroring filling, the small circle tissue is lost in Figure 6(c). The images reconstructed by POCS-TV choosing zero image, FBP image (Figure 6(b)), and our optimized image (Figure 6(c)) separately are shown in Figure 7. Reconstructions (Figures 7(a) and 7(b)) from POCS-TV with initial zero image and FBP image produce unsatisfactory results where the artifacts are not removed effectively. In Figure 7(c), POCS-TV with our optimized initial estimation (small circle tissue is lost) can reconstruct artifacts-free image. The lost small circle tissue by mirroring filling in our method can be recovered drastically.

**Figure 8:**
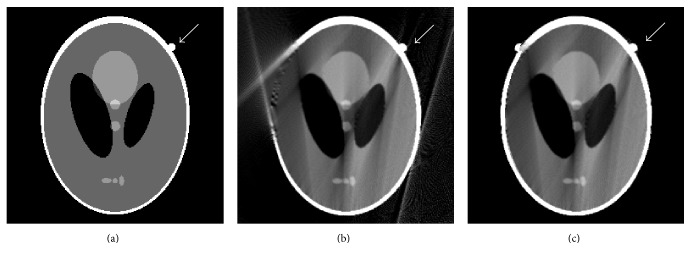
Modified Shepp-Logan phantom and initially reconstructed images: (a) modified phantom, (b) reconstructed image by FBP, and (c) reconstructed image using the proposed algorithm.

**Figure 9:**
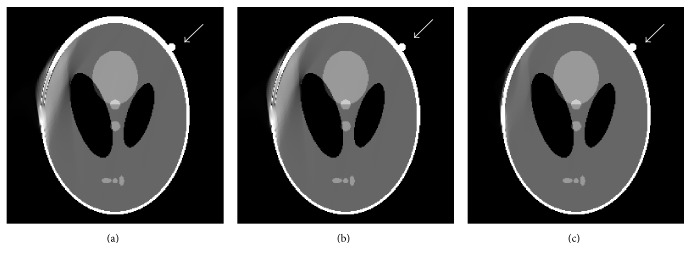
Reconstruction results by different initial images: (a) result from POCS-TV with initial zero image, (b) result from POCS-TV with initial FBP image, and (c) result from POCS-TV with proposed initial image.

Another modified Shepp-Logan is shown in Figure 8(a). One small circle tissue indicated is included. Initial images reconstructed by conventional FBP and our proposed method are shown in Figures 8(b) and 8(c), respectively. By mirroring filling, the other small circle tissue appears in Figure 8(c). POCS-TV with initial zero image and FBP image reconstruct results with conspicuous local artifacts, shown in Figures 9(a) and 9(b). POCS-TV with our optimized initial estimation (new small circle tissue appears) generates artifacts-free image, and the new small circle tissue caused by mirroring filling in our method can be removed effectively.

From the reconstructions for modified Shepp-Logan phantom, we can see that, although the scanned object is not strictly axis-symmetrical, our proposed algorithm is able to show more advantages for limited-angle CT.

### 3.2. Real Data Experiment

**Figure 10:**
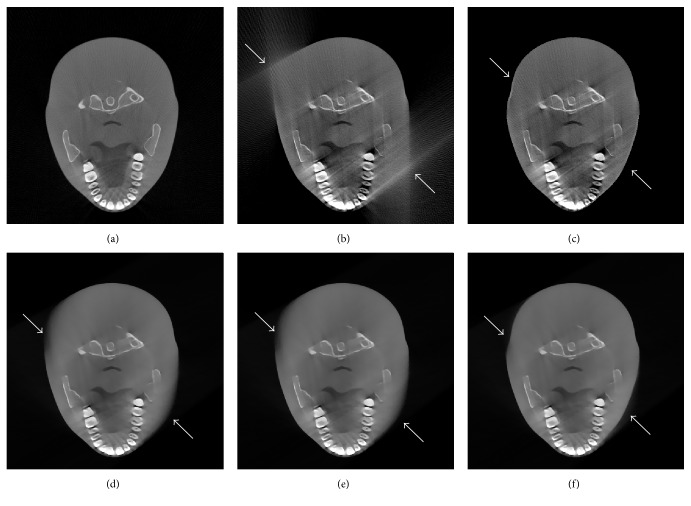
Different initial images and iterative reconstruction results of real head phantom: (a) reference image, (b) initial image by FBP, (c) initial image by proposed algorithm, (d) result from POCS-TV with initial zero image, (e) result from POCS-TV with initial FBP image, and (f) result from POCS-TV with proposed initial image.

To evaluate the performance of proposed algorithm for X-ray CT, reconstruction using real CT projection data is tested. Single circle scan and fan-beam imaging geometry are used to obtain the projections in our developed laboratory CT scanner. The distance between X-ray source and rotation axis is 106.61 cm and the distance between detector and rotation axis is 52.36 cm. The initial position of X-ray source is located in −*y*-axis. The scanned object is a head phantom. 120 projections are equally captured over a 120-degree range to perform limited-angle CT reconstruction. Reconstruction from 360 projections in a circle is considered reference true image, shown in Figure 10(a). Initial images reconstructed by conventional FBP and our proposed method are shown in Figures 10(b) and 10(c), respectively. Images reconstructed by POCS-TV using different initializations are shown in Figures 10(d)–10(f). We have observed that our method can lead to better results.

## 4. Discussion and Conclusion

In this work, we propose an initialization procedure for limited-angle CT iterative image reconstruction when an object's outer contour is roughly axis-symmetrical. The proposed method makes full use of the image symmetry to eliminate the deformation artifacts and supply POCS-TV with good initial image. For numerical experiments, compared with zero image and FBP image as initial images for POCS-TV, results by our method are better with 92% (0.0002 < 0.0024) gains in terms of MSE measure and with 46% (22.99 dB > 12.42 dB) gains in terms of SNR measure. The results of the real head phantom also validate the superiority of the proposed method.

The POCS-TV algorithm was implemented effectively using the widely utilized OSL iteration scheme. However, global convergence is an open issue as many existing OSL algorithms. POCS-TV is lack of strict global convergence proof. As presented in reconstructed images and the cost figures, poorly initialized POCS-TV gets locked into a local minimum (MSE) and a local maximum (SNR), and the outcome could not be improved apparently by further iterations. Meanwhile, well initialized POCS-TV performs effectively in practice although the global convergence cannot be guaranteed. In this paper, we studied the results based on visualization of reconstructed images, maximum of SNR, and minimum of MSE. Obviously, more theoretical insight of POCS-TV convergence issue is necessary in the future.

The position of artifacts varies due to the different arch explored by the focal spot. It is true that our proposed method would be useless if the sampled arch is centered on the object axis of symmetry. However, satisfactory images reconstructed by POCS-TV with poor initialization (zero image and FBP image) cannot be generated in this case. Thus, instead of letting the sampled arch centered on the object axis of symmetry, one can decide the side of the body to be explored and reconstruct initial image with less artifacts using the prior information of object symmetry.

3D FDK algorithm, as an extension to 2D FBP algorithm, has been widely used in CBCT reconstruction. In the limited-angle scanning case, the artifacts of 3D reconstruction using FDK are as almost the same as those of 2D FBP. Our proposed initialization can be promisingly applied to 3D reconstruction where FDK algorithm combined with object symmetry property is used and then POCS-TV in 3D case is performed.

In this work, for the modified Shepp-Logan phantom, we tested the case of a small asymmetry to show that our method can produce satisfactory results. On top of this, another test for real head phantom was done when the symmetry axis of object does not exactly match the scanner axis (namely, the *y*-axis). This case is often caused by positioning errors, hardly avoided in clinical practice. However, under the condition of a big positioning error, it is necessary to redetermine the object symmetry axis of image reconstructed by FBP using some basic image processing technology such as scale-invariant feature transform. Thus, symmetry in respect to a scanner axis (namely, the *y*-axis) is not a mandatory feature. As long as the sampled arch is not centered on the object axis of symmetry, the proposed method in this study may have benefit. Moreover, the computation time is an important factor that restrains the application of iterative methods into practice. With the fast development and increasing capability of graphic processing units (GPU) [22–24] in medical image processing, under the limited-angle cone-beam CT (CBCT) scan mode, it is another key research to reconstruct CBCT images accurately and fast using GPU acceleration.
